# Local discrepancies in measles vaccination opportunities: results of population-based surveys in Sub-Saharan Africa

**DOI:** 10.1186/1471-2458-14-193

**Published:** 2014-02-21

**Authors:** Lise Grout, Nolwenn Conan, Aitana Juan Giner, Northan Hurtado, Florence Fermon, Alexandra N’Goran, Emmanuel Grellety, Andrea Minetti, Klaudia Porten, Rebecca F Grais

**Affiliations:** 1 Epicentre, 8, rue St Sabin, 75011 Paris, France; 2 Médecins Sans Frontières, 8, rue St Sabin, 75011 Paris, France

**Keywords:** Measles, Immunization, Expanded program of immunization, Supplemental, Immunization activity, Outbreak response immunization, Vaccine coverage

## Abstract

**Background:**

The World Health Organization recommends African children receive two doses of measles containing vaccine (MCV) through routine programs or supplemental immunization activities (SIA). Moreover, children have an additional opportunity to receive MCV through outbreak response immunization (ORI) mass campaigns in certain contexts. Here, we present the results of MCV coverage by dose estimated through surveys conducted after outbreak response in diverse settings in Sub-Saharan Africa.

**Methods:**

We included 24 household-based surveys conducted in six countries after a non-selective mass vaccination campaign. In the majority (22/24), the survey sample was selected using probability proportional to size cluster-based sampling. Others used Lot Quality Assurance Sampling.

**Results:**

In total, data were collected on 60,895 children from 2005 to 2011. Routine coverage varied between countries (>95% in Malawi and Kirundo province (Burundi) while <35% in N’Djamena (Chad) in 2005), within a country and over time. SIA coverage was <75% in most settings. ORI coverage ranged from >95% in Malawi to 71.4% [95% CI: 68.9-73.8] in N’Djamena (Chad) in 2005.

In five sites, >5% of children remained unvaccinated after several opportunities. Conversely, in Malawi and DRC, over half of the children eligible for the last SIA received a third dose of MCV.

**Conclusions:**

Control pre-elimination targets were still not reached, contributing to the occurrence of repeated measles outbreak in the Sub-Saharan African countries reported here. Although children receiving a dose of MCV through outbreak response benefit from the intervention, ensuring that programs effectively target hard to reach children remains the cornerstone of measles control.

## Background

The World Health Organization (WHO) comprehensive measles control strategy aims to reduce global measles mortality by at least 95% by the end of 2015 compared with 2000 estimates and achieve measles elimination in at least five WHO regions by the end of 2020. One of the components of the strategy is to achieve and maintain high levels of population immunity by providing high vaccination coverage with two doses of measles-containing vaccine (MCV) [1]. Children should receive the first MCV dose through the Expanded Program of Immunization (EPI). A second should be delivered either through periodic supplemental immunization activities (SIAs) or through EPI.

Moreover, since 2009, outbreak response immunization (ORI) is part of WHO strategy for response to measles outbreaks in measles mortality reduction settings [2]. If assessment indicates the risk of a large outbreak, non-selective reactive mass vaccination campaign should be implemented as soon as the outbreak is confirmed. The aim is to reduce morbidity and mortality in the immediate by delivering at least one dose of MCV to susceptible children.

Although measles mortality was reduced by 91% between 2001 and 2008 in the WHO African region countries [3], measles remains a leading cause of vaccine-preventable deaths in Africa [4]. Failure to deliver at least one dose of MCV remains the primary reason for high measles mortality.

In order to sustain gains in measles control and further reduce mortality, an understanding of whether each of these vaccination opportunities reaches children is essential. Vaccination coverage is the key indicator to measure the progress of measles control. Ministries of Health (MoH) report annual administrative coverage (i.e. number of doses delivered divided by estimated number of children in the targeted age group) based on population estimates. In many contexts, however, population estimates, are often not up-to-date (i.e. population censuses might not be frequently performed and vital event registration may be absent or partial), resulting in biased or inaccurate estimates. Consequently, population-based surveys are often the best available means to estimate vaccination coverage at both local and national levels.

Over the past eight years, the non-governmental organization Médecins Sans Frontières (MSF) supported MoH of several countries in sub-Saharan Africa in the implementation of ORI through non-selective mass vaccination campaigns. Following these campaigns and as part of the overall response to measles epidemics, punctual household-based measles coverage surveys were conducted [5-9]. The main objective of these surveys was to estimate vaccination coverage through ORI. These surveys also aimed to estimate measles vaccine coverage through previous vaccination opportunities: in EPI, SIA, or outbreak response campaigns. Here, we present a secondary data analysis of these different surveys to describe measles vaccine coverage through different vaccination opportunities in different settings in sub-Saharan African countries. The aim is to provide additional information on whether these different vaccination opportunities reach children in need.

## Methods

We considered all household-based measles vaccination coverage surveys conducted by Epicentre after a non-selective mass vaccination campaign that evaluated different opportunities and for which the complete dataset was available. We defined a survey as a unique analysis of measles vaccination coverage. We considered a total of 24 surveys conducted between 2005 and 2011 in 23 sites in 6 countries (Chad, Central African Republic (CAR), Democratic Republic of Congo (DRC), Cameroon, Malawi and Burundi). Surveys were conducted in different locations within a country in Chad, DRC, Malawi and CAR. Two surveys were conducted at two different times in N’Djaména (Chad). A total of 13 of the study sites were considered as urban or a mix of urban-rural (N’djamena city, Matadi city, Mbuji-Mayi city, Blantyre, Lilongwe, Mzimba and Balaka/Machinga districts, Likasi and Lubumbashi cities and Kabo city), the others were considered as rural.

### Design and settings of vaccine coverage studies

Surveys were conducted on average 30 days after the first day of the mass vaccination campaign (range: 1 day to 4 months). The age group included in the surveys varied according to the age group targeted by the ORI. All surveys employed probability proportional to size cluster-based sampling (Additional file 1: Table S1), except the surveys conducted in N’Djamena (Chad) in 2005 and 2010 which used Lot Quality Assurance Sampling. The first household to be surveyed in each cluster or lot was selected randomly by spatial-based sampling [8], GPS-based sampling, complete household enumeration or by the EPI method [10]. Method for selection of the first household was by preference by complete enumeration when logistical and security constraints allowed. Subsequent households were selected by proximity. Sample size varied according to the design and hypothesis about existing vaccination coverage (range: 942 to 6622 children).

### Data collection

Trained surveyors conducted face-to-face interviews with child’s main caregiver. Standardized, pre-piloted questionnaires were used and interviews were conducted in local language(s). Information was collected on demographic characteristics (age at the time of the survey or date of birth, sex) and vaccination status for different vaccine opportunities (Additional file 1: Table S1). When possible, information on vaccination history was verified with vaccination card. When card was not available, probing questions, such as place were the vaccination took place or injection site, were asked to minimize the risk of misclassification.

Vaccination status was collected for different opportunities. ORI implemented by MoH with the support of MSF (MSF/MoH ORI) and EPI were considered in all study sites. Although different SIAs had been implemented at different time points in these countries, only vaccination during the most recent was considered. Consequently, we considered SIA implemented in 2002 for the survey conducted in Mbuji-Mayi (DRC) in 2006; SIA implemented in 2008 for the surveys conducted in Malawi in 2010; and, SIA conducted in 2007 for the surveys conducted in DRC in 2011. In Maroua (Cameroon) and Kirundu (Burundi), SIAs were implemented before the ORI but were not included in the surveys. Lastly, in four countries (Cameroon, Malawi, Chad and CAR), we also evaluated MoH mass campaigns in response to outbreaks conducted before MSF/MoH ORI.

Administrative coverage was provided by local health authorities.

### Data analysis

Children were considered as vaccinated through a specific opportunity if they were eligible for vaccination (i.e. in the target age group) and vaccination was reported by card examination or oral history.

Vaccine coverage and 95% confidence interval (95% CI) were estimated for each vaccine opportunity. EPI coverage was defined as the proportion of children vaccinated through the routine program among children aged 9 to 11 months (i.e. in the targeted window for this program) and 12 to 23 months (i. e. who should have recently received their MCV through EPI). SIA, ORI and other potential MoH reactive campaigns coverage were defined as the proportion of children vaccinated amongst the target age group at the time of the campaign. ORI coverage was calculated by age group.

If the delay between the last day of the MoH/MSF campaign and the first day of the vaccine coverage survey was <31 days, age at the time of the survey was considered to be similar to age at the time of the MoH/MSF campaign. If not, age at the first day of the MoH/MSF campaign was calculated.

To evaluate if children received the two recommended doses, we calculated the number of doses received by summing the number of occasions a child was considered vaccinated. Only children with all information on vaccine opportunities were included in this sub-analysis, otherwise number of doses was considered as missing. In Malawi and DRC, where EPI, SIA and ORI opportunities were evaluated, we estimated the proportion of children vaccinated through ORI according to the number of doses of MCV they received before, and the proportion of children for whom the ORI dose represent a first, a second or a third dose of MCV. For this sub-analysis, information on EPI vaccination was considered for all children aged 9 month or older included in the survey and not only for children 9 to 23 months of age.

To explore potential associations between vaccination coverage and explanatory variables, Poisson regression was conducted when several places were surveyed at the same time. Wald tests were used to test explanatory variables (p < 0.05). Estimations were weighed to account for survey designs. Data was entered in EpiData v3 (Odense, Denmark) and analysed with Stata v11 (College Station, Texas, USA).

### Ethical considerations

Surveys were conducted as part of the monitoring and evaluation of the emergency response and were not submitted for formal ethical approval in the country of the study. The MSF Ethical Review Board exempts such surveys from review as they constitute part of the emergency response and use a standard and widely accepted survey protocol. All studies, however, were approved by local and national authorities through the convocation of exceptional meetings. In N’Djamena, surveys were conducted with the authorization of the national technical committee for the battle against epidemics; in Matadi and Mbuji Mayi (DRC), authorization was obtained from the Ministry of Health; in Maroua (Cameroon), a request was made to the Division of Operational Research within the Ministry of Health for specific ethical clearance for the survey and was granted; in Malawi, the study was implemented in collaboration with the MoH after obtaining permission to carry out the survey, in Katanga, authorization was delivered by national and provincial health authorities.

The main caregivers of all participants provided oral informed consent via a specific explanation in the local language as well as a written document providing information on the use of information and on confidentially. No specific ethnic or identifying information was recorded and all participants were free to refuse participation in the surveys. Vaccination and care were provided free of charge irrespective of participation in surveys.

## Results

Between 2005 and 2011 vaccination data was collected from 60,895 children living in six countries. Half of them were male (sex ratio = 1.0). Twelve of the surveys included children 6 months to 14 years old, 5 surveys included children 6 months to 4 years old and 1 considered children 6 months to 9 years old.

### ORI coverage

Overall, MoH/MSF campaign coverage was >90% in most surveys conducted in DRC (except Malemba-Nkulu, Kambove and Kapolowe), Malawi (except Mzimba) and Burundi (Table 1). Coverage was >95% in 3 districts of DRC, including two urban districts (Likasi and Lubumbashi) and one rural district (Kipushi). Coverage was also >95% in two urban-rural districts of Malawi (Chiradzulu and Thiolo). The lowest MoH/MSF campaign coverage was estimated in N’Djaména (Chad) in the survey conducted in 2005 where less than 75% of children were vaccinated.

**Table 1:** Outbreak response immunization coverage estimates (%) with 95% confidence interval by age group and study site, 2006-2011

**Survey**				**All targeted ages**		**6 - 8 months**		**9 - 11 months**		**12 - 24 months**		**25 - 59 months**		**5 - 9 years**		**10 - 14 years**	
**Country**	**Location**	**Year**	**Target**	**VC %**	**CI 95%**	**VC %**	**CI 95%**	**VC %**	**CI 95%**	**VC %**	**CI 95%**	**VC %**	**CI 95%**	**VC %**	**CI 95%**	**VC %**	**CI 95%**
Chad	N’Djamena	2005	6-59 m	71.4	[68.9;73.8]	78.6	[63.8;88.4]	69.8	[54.6;81.6]	70.5	[65.2;75.3]	71.4	[68.3;74.4]	-		-	
DRC	Matadi	2006	6 m-15 y	97.1	[94.0;98.6]	93.1	[82.7;97.5]	93.7	[81.0;98.1]	95.8	[91.1;98.0]	96.9	[93.3;98.6]	97.5	[93.5;99.1]	98.6	[96.1;99.5]
	Mbuji Mayi	2006	6-59 m	94.3	[93.2;95.3]	94.4	[89.4;97.1]	94.3	[91.1;96.4]	93.8	[91.6;95.4]	94.5	[93.4;95.5]				
Cameroon	Maroua	2009	9 m-15 y	80.3	[76.7;83.5]	-		61.3	[51.2;70.5]	77.0	[69.6;83.0]	79.4	[74.5;83.6]	84.7	[80.9;87.8]	79.1	[74.7;82.9]
Chad	N’Djamena	2005	6 m-15 y	84.9	[83.2;86.5]	70.1	[60.3;78.4]			78.5	[72.1;83.8]	84.9	[81.7;87.5]	87.8	[85.6;89.7]	-	
Malawi	Blantyre	2010	6 m-15 y	93.1	[90.6;95.0]	90.7	[75.9;96.8]	84.3	[69.8;92.5]	87.9	[81.3;92.4]	92.1	[89.1;94.3]	95.2	[92.3;97.0]	94.0	[89.9;96.5]
	Chiradzulu	2010	6 m-15 y	98.0	[97.3;98.5]	91.0	[79.9.96.2]	92.2	[80.8;97.0]	98.2	[95.1;99.4]	97.9	[96.5;98.8]	98.5	[97.6;99.1]	98.0	[96.1;99.0]
	Thyolo	2010	6 m-15 y	96.7	[95.6;97.5]	75.3	[61.0;85.6]	84.8	[71.2;92.6]	96.9	[94.3;98.3]	96.6	[94.1;98.1]	98.2	[96.8;99.0]	97.4	[95.8;98.4]
	Mangochi	2010	6 m-15 y	96.5	[94.7;97.7]	93.0	[84.6;97.0]	95.2	[88.0;98.2]	95.3	[92.2;97.2]	97.3	[95.4;98.4]	96.7	[94.2;98.1]	96.2	[93.9;97.7]
	Lilongwe	2010	6 m-15 y	95.8	[94.2;96.9]	91.5	[80.1;96.7]	88.9	[80.1;94.0]	91.4	[86.5;94.6]	95.2	[92.6;96.9]	97.3	[95.2;98.4]	97.0	[94.8;98.2]
	Mzimba	2010	6 m-15 y	92.3	[89.6;94.4]	82.1	[71.9;89.2]	92.5	[84.7;96.5]	95.5	[91.8;97.5]	90.7	[87.9;93.0]	93.9	[90.6;96.0]	92.2	[88.1;94.9]
	Balaka – Machinga	2010	6 m-15 y	96.0	[94.1;97.4]	93.7	[85.3;97.5]	96.7	[90.0;99.0]	95.3	[90.5;97.7]	95.8	[92.9;97.6]	97.1	[95.0;98.3]	95.2	[92.2;97.0]
	*Overall*	*2010*	*6 m-15 y*	*95.5*	*[94.9;96.1]*	*86.3*	*[81.6;89.9]*	*89.9*	*[85.8;92.8]*	*94.3*	*[92.9;95.5]*	*95.2*	*[94.3;96.0]*	*96.9*	*[96.2;97.5]*	*95.9*	*[95.0;96.7]*
DRC	Likasi city	2011	6 m-15 y	99.3	[98.9;99.6]	95.7	[88.5;98.5]	99.4	[95.9;99.9]	98.3	[96.3;99.2]	99.3	[98.4;99.7]	99.6	[99.0;99.9]	99.8	[99.2;99.9]
	Lubumbashi	2011	6 m-15 y	98.1	[97.3;98.6]	97.6	[92.4;99.3]	96.3	[88.8;98.9]	97.9	[95.9;98.9]	97.9	[96.5;98.7]	98.1	[96.8;98.8]	98.6	[97.6;99.2]
	Kapolowe	2011	6 m-15 y	97.0	[88.0;99.3]	100.0	-	93.3	[61.6;99.2]	96.9	[86.6;99.4]	96.9	[85.9;99.4]	96.9	[89.4;99.2]	97.2	[86.9;99.5]
	Kambove	2011	6 m-15 y	94.7	[81.8;98.6]	93.7	[63.0;99.2]	97.8	[84.3;99.7]	89.8	[71.3;96.9]	95.1	[76.8;99.1]	96.0	[83.6;99.1]	93.9	[77.2;98.6]
	Kasenga	2011	6 m-15 y	93.8	[91.0;95.7]	91.9	[82.8;96.4]	90.3	[74.4;96.8]	91.0	[83.5;95.2]	91.8	[87.2;94.8]	94.7	[91.9;96.5]	96.8	[93.1;98.6]
	Malemba-Nkulu	2011	6 m-10y	81.1	[76.5;84.9]	75.1	[61.2;85.2]	78.0	[64.1;87.6]	83.5	[77.0;88.5]	79.9	[74.6;84.2]	82.2	[76.8;86.6]	-	
	Kipushi	2011	6 m-15 y	97.2	[95.7;98.2]	89.6	[70.4;96.9]	93.3	[74.2;98.6]	97.0	[92.9;98.8]	96.7	[94.3;98.1]	98.4	[96.8;99.2]	97.5	[94.7;98.9]
	*Overall*	*2011*	*6 m-15 y*	*96.7*	*[95.9;97.4]*	*95.0*	*[92.3;96.8]*	*94.6*	*[90.9;96.8]*	*96.0*	*[94.5;97.2]*	*96.2*	*[95.2;97.0]*	*96.7*	*[95.7;97.5]*	*98.2*	*[97.2;98.9]*
Burundi	Kirundo	2011	6 m-15 y	96.7	[94.4;98.1]	93.9	[63.7;99;3]	84.7	[55.7;96.1]	93.4	[85.4;97.2]	97.5	[94.1;99.0]	97.3	[94.9;98.6]	97.2	[93.3;98.9]
Chad	Moissala (1)	2011	9-59 m	84.3	[75.8;90.2]	-		73.5	[59.2;84.1]	84.3	[75.3.90.4]	85.4	[76.5;91.3]	-		-	
	Moissala (2)	2011	6 m-15 y	94.0	[83.8;98.0]	-		79.7	[62.6;90.2]	94.9	[77.2;99.0]	95.2	[86.8;98.3]	-		-	
	*Overall*	*2011*	*6 m-15 y*	*86.4*	*[79.8;91.1]*	*-*		*74.8*	*[63.5;83.6]*	*86.6*	*[79.5;91.5]*	*87.5*	*[80.7;92.2]*	*-*		*-*	
CAR	Kabo	2011	9 m-15 y	92.0	[85.5;95.7]	80.0	[60.5;91.3]	95.4	[85.3;98.7]	92.9	[83.8;97.0]	92.6	[85.4;96.4]	90.3	[82.5;94.9]	93.4	[84.4;97.3]
	Batangafo	2011	9 m-15 y	90.0	[88.5;91.3]	-		88.7	[78.0;94.6]	89.0	[84.8;92.2]	89.6	[87.3;91.6]	91.4	[89.1;93.3]	88.1	[84.2;91.2]

VC: Vaccine coverage; 95% CI: 95% confidence interval; DRC: Democratic Republic of Congo; CAR: Central African Republic.

Estimated coverage varied within locations in Malawi (p = 0.004) and in DRC in 2011 (p < 0.001), but not in Moïssala district (Chad) (p = 0.07). In Malawi, the percentage of children vaccinated was highest in the rural area of Chiradzulu and lowest in the urban-rural district of Mzimba. In Katanga province (DRC), ORI 2011 campaign coverage varied from 99.3% in the urban zone of Likasi city to 81.1% in the rural northern district of Malemba-Nkulu. ORI coverage was significantly lower among children <1 year-old in the surveys conducted in N’Djaména (Chad) in 2010, in Maroua (Cameroon) and in Thyolo district (Malawi). ORI coverage did not vary by sex.

### EPI coverage

The surveys conducted in N’Djaména (Chad) in 2005 showed the lowest estimate with only around one third of children 12 to 23 months of age receiving MCV through EPI (Table 2). Burundi had the highest estimates with vaccination coverage above 90%. EPI coverage was close to 90% in all districts of Malawi. EPI coverage was significantly lower among children aged 9 to 11 than amongst 12-23 months old in Katanga province (DRC) (p = 0.01), Malawi (p < 0.001) and Moissala district (Chad) (p < 0.001).

**Table 2:** Expanded Program for Immunization (EPI) coverage estimates (%) with 95% confidence interval, children 9 to 11 and 12 to 23 months old, by study site, 2006-2011

**Survey**			**EPI estimates**				**Administrative coverage**		
			**9 to 11 months**		**12 to 23 months**				
**Country**	**Location**	**Year**	**VC %**	**CI 95%**	**VC %**	**CI 95%**	**CV %**	**Area**	**Year**
Chad	N’Djamena	2005	21.3	[13.5;32.0]	31.3	[27.2;35.7]	48%	National	2005
DRC	Matadi	2006	79.4	[66.4;88.2]	87.7	[81.0;92.2]	71%	National	2005
	Mbuji Mayi	2006	50.7	[44.7;56.8]	71.4	[67.1;75.3]	67.2%	Mbuji-Mayi	2005
Cameroon	Maroua	2009	65.9	[50.7;78.3]	66.1	[58.5;73.0]	89%	City	2008
Chad	N’Djamena	2010	30.9	[22.6;40.8]	68.6	[61.7;74.7	72%	National	2009
Malawi	Blantyre	2010	37.3	[25.2;51.3]	97.9	[95.1;99.1]	92%	National	2009
	Chiradzulu	2010	59.6	[44.9;72.8]	98.2	[95.1;99.4]			
	Thyolo	2010	51.8	[37.8;65.6]	95.5	[92.0;97.6]			
	Mangochi	2010	54.3	[43.7;64.5]	91.3	[87.4;94.0]			
	Lilongwe	2010	74.4	[63.4;83.0]	93.6	[87.2;96.9]			
	Mzimba	2010	53.6	[43.7;63.3]	90.9	[86.9;93.7]			
	Balaka - Machinga	2010	78.3	[64.5;87.7]	97.6	[95.1;98.8]			
	*Overall*	*2010*	*57.7*	*[52.3;62.9]*	*95.0*	*[93.6;96.0]*			
DRC	Likasi city	2011	58.4	[47.6;68.5]	85.9	[80.9;89.7]	96% to 129%	City	2010
	Lubumbashi city	2011	51.4	[37.8;64.8]	76.6	[69.6;82.5]	57% to 153%	City	2010
	Kapolowe	2011	76.5	[50.1;91.3]	69.0	[55.2;80.1]	73%	Health zone	2010
	Kambove	2011	74.6	[46.9;90.7]	61.9	[46.9;74.9]	90%	Health zone	2010
	Kasenga	2011	75.5	[55.3;88.5]	77.6	[69.5;84.0]	103%	Health zone	2010
	Malemba-Nkulu	2011	61.7	[42.7;77.7]	76.1	[68.2;82.4]	90%	Health zone	2010
	Kipushi	2011	91.6	[69.5;98.1]	83.8	[74.0;90.3]	78%	Health zone	2010
	*Overall*	*2011*	*57.6*	*[48.4;66.2]*	*76.4*	*[72.1;80.2]*	***-***		
Burundi	Kirundo	2011	83.4	[58.4;94.7]	99.1	[93.1;99.9]	88%	National	2010
Chad	Moissala (1)	2011	54.9	[38.8;70.0]	73.5	[64.2;81.0]	87%	National	2009
	Moissala (2)	2011	32.8	[23.2;44.3]	71.6	[61.0;80.2]			
	*Overall*	*2011*	*49.1*	*[37.2;61.2]*	*73.1*	*[65.5;79.4]*			
CAR	Kabo	2011	17.0	[5.6;41.3]	63.7	[48.1;76.9]	62.5%	Batangafo	2010
	Batangafo	2011	46.2	[33.2;59.7]	72.7	[66.4;78.3]	62%	National	2009

VC: Vaccine coverage; 95% CI: 95% confidence interval; DRC: Democratic Republic of Congo; CAR: Central African Republic.

Surveys conducted in diverse locations of the same country showed significant differences in Malawi (p < 0.001), and in DRC (p = 0.005), but not in Moïssala district (Chad) (p = 0.76). In N’Djaména (Chad) there was a significant increase in vaccination coverage from 2005 to 2010.

Administrative coverage overestimated survey results in Chad (N’Djaména 2005 and Moïssala 2011), Cameroon and several health zones of DRC (Likasi, Kambove, Kasenga and Malemba-Nkulu). The surveys conducted in Matadi (DRC), Kirundo (Burundi) and Batangafo (CAR) showed higher vaccination coverage than administrative estimates. Administrative coverage best approximated survey results in Malawi.

### SIA coverage

The surveys conducted in Katanga district in DRC in 2011 showed the highest coverage estimates for SIA activities (Table 3). Among these, the greatest coverage was obtained in Lubumbashi city and the lowest in Kambove, both in DRC. In Malawi, SIA coverage also varied within the country. Reported SIA administrative coverage was typically very high and often above 95%.

**Table 3:** Supplemental Immunization Activities (SIA) coverage estimates (%) with 95% confidence interval, for the most recent SIA before the outbreak response immunization, by study site, 2006-2011

**Survey**			**SIA**		**SIA estimates**		**SIA administrative coverage**
**Country**	**Location**	**Year**	**Year**	**Target pop.**	**VC %**	**CI 95%**	**VC %**
Chad	N’Djamena	2005	-	-	-		-
DRC	Matadi	2006	-	-	-		-
	Mbuji Mayi	2006	2002	6 m - 15 y	49.5	[42.5;56.5]	96.4
Cameroon	Maroua	2009	2006	9 - 59 m	*NA*		94
Chad	N’Djamena	2010	-	-	-		-
Malawi	Blantyre	2010	2008	9 - 59 m	46.8	[39.6;54.1]	99
	Chiradzulu	2010			57.4	[51.6;63.0]	
	Thyolo	2010			69.1	[63.7;74.1]	
	Mangochi	2010			62.8	[57.3;67.9]	
	Lilongwe	2010			59.9	[54.8;64.8]	
	Mzimba	2010			47.0	[41.2;53.0]	
	Balaka - Machinga	2010			68.6	[63.1;73.6]	
	*Overall*	*2010*			*60.7*	*[58.3;63.1]*	
DRC	Likasi city	2011	2007	6-59 m	82.6	[77.6;86.6]	101-112
	Lubumbashi city	2011			89.1	[85.2;92.0]	85-142
	Kapolowe	2011			81.8	[70.4;89.4]	119
	Kambove	2011			68.2	[49.4;82.5]	95
	Kasenga	2011			85.5	[80.4;89.5]	96
	Malemba-Nkulu	2011			77.3	[70.6;82.9]	95
	Kipushi	2011			84.5	[78.0;89.4]	94
	*Overall*	*2011*			*85.5*	*[82.7;87.8]*	*-*
Burundi	Kirundo	2011	2009	9-59 m	*NA*		95
Chad	Moissala (1)	2011	2006		*NA*		-
	Moissala (2)	2011	2006		*NA*		-
CAR	Kabo	2011	2008		*NA*		-
	Batangafo	2011	2008		*NA*		-

NA: Not assessed; VC: Vaccine coverage; 95% CI: 95% confidence interval; DRC: Democratic Republic of Congo; CAR: Central African Republic.

### Other mass vaccination campaigns

Surveys results showed that previous ORI conducted by MoH had the highest coverage estimate in CAR in 2011 with 79.0% [95% CI: 75.7.4-82.0%] in Batangafo and 71.0% [95% CI: 58.4-80.6%] in Kabo (Table 4). Estimated coverage was <55% in all the other locations and reached a lowest 26.7% [95% CI: 21.4-32.8%] in Maroua (Cameroon). Official administrative coverage was unavailable for these locations at this spatial scale.

**Table 4:** Previous outbreak response immunization coverage estimates (%) with 95% confidence interval, by study site, 2006-2011

**Survey**			**Previous ORI**		**Coverage estimates**	
**Country**	**Location**	**Year**	**Year**	**Target population**	**VC %**	**CI 95%**
Cameroon	Maroua	2009	2008	9 - 59 m	26.7	[21.4;32.8]
Chad	N’Djamena	2010	2009		53.2	[51.0;55.3]
Malawi	Blantyre	2010	2010	9 - 59 m	42.5	[34.4;51.2]
	Chiradzulu	2010	-		-	
	Thyolo	2010	-		-	
	Mangochi	2010	-		-	
	Lilongwe	2010	2010	9 - 59 m	34.0	[22.4;47.8]
	Mzimba	2010	2010	9 - 59 m	29.4	[22.5;37.4]
	Balaka - Machinga	2010	-		-	
CAR	Kabo	2011	2011	9-47 m	71.0	[59.4;80.4]
	Batangafo	2011	2011	9-47 m	79.0	[75.7;82.0]

VC: Vaccine coverage; 95% CI: 95% confidence interval; CAR: Central African Republic.

### MCV vaccination in different opportunities

While taking into account the different vaccination opportunities, a portion of children remained unvaccinated. The proportion of unvaccinated children was highest in N’Djaména in 2005 where 16.8% [95% CI: 15.0%-18.6%] of children remained unvaccinated. This figure decreased to 5.6% [95% CI: 4.6%-6.7%] in the survey conducted in 2010. However, the proportion of unvaccinated children was >5% in several sites: Moissala (Chad) (9.9% [95% CI: 5.7-16.7%]), Kabo (CAR) (8.7% [95% CI: 5.3-13.9%]), Malemba-Nkulu (DRC, 2011) (8.1% [95% CI: 5.6-11.6%]) and in Maroua city (Cameroon) (6.5% [95% CI: 4.7%-9.0%]).

After the ORI campaign, three-quarters of the children living in N’Djamena in 2005 had only received one dose of MCV (75.4% [95% CI: 73.4-77.3%]). This proportion was around one-third in Moïssala (Chad) (32.2% [95% CI: 25.9-39.2%]) and Maraoua (Cameroon) (31.9% [95% CI: 28.3-35.9%]) and between one-quarter and one-third in Kabo (29.7% [95% CI: 21.2-39.8%]), Batangafo (28.4% [95% CI: 26.2-30.8%]), and Mbuji-Mayi 27.3% [95% CI: 24.9-29.9%].

The surveys conducted in Malawi in 2010 and in DRC in 2011 where children had previous vaccination opportunities through EPI and SIA showed that ORI provided the first MCV dose to approximately 90% of children 6 to 8 months old. Among children in the EPI target group (9 to 11 months), approximately one-third received their first dose of MCV during the ORI and half received their second dose. The highest estimate for the second dose of MCV during ORI for children 9 to 11 months old was in Malawi with 55.8% [95% CI: 50.1-61.3%]. The majority of children older than 1 year but too young to be eligible for the last SIA received a second dose during the ORI. This was 78.0% of the children [95% CI: 75.4-80.4%] in Katanga province (DRC) and up to 90.9% [95% CI: 89.3-92.2%] in Malawi.

In these contexts, over half of the children eligible for the last SIA received a third dose of MCV. This was 55.8% [95% CI: 53.5-58.2% in the surveys conducted in Katanga province (DRC) and 63.4% [95% CI: 59.7-66.9%] in the surveys conducted in Malawi.

However, the proportion of children vaccinated during ORI was significantly higher among children who previously received at least one dose of MCV (Malawi and Katanga province, p < 0.001). This is best illustrated in the rural northern zone of Katanga Province (DRC) where 52.3% [95% CI:42.6%;61.9%] of children never vaccinated before the ORI, 89.9% [95% CI:86.0%;92.7%] of children who received 1 dose of MCV prior to the ORI and 91.1% [95% CI:86.8%;94.1%] of those who received 2 doses prior to the ORI were vaccinated during the campaign.

## Discussion

We present the results of 24 population-based measles vaccination coverage surveys conducted in between 2005 and 2011 in 28 locations in Sub-Saharan Africa. In 2008, the WHO African Region measles technical advisory group recommended establishing a measles pre-elimination goal, to be achieved by the end of 2012, with the following immunization targets: >90% national MCV routine coverage, with at least 80% coverage in all districts; and ≥95% SIA coverage in all districts [11]. Routine vaccination coverage reached high levels in several districts of Malawi and in Kirundu (Burundi), but most of the surveys showed insufficient EPI coverage.

Survey results showed that the highest vaccination coverage was obtained through ORI. For most infants 6 to 8 months of age, ORI provided the first MCV vaccination. These infants are at higher risk of complications and death in case of illness highlighting the effectiveness of vaccinating this population in ORI.

EPI coverage appeared to be lower among children aged 9 to 11 months than 12 to 23 months old. This might show a delay in the age of routine vaccination. The recommendation to enlarge the targeted age group for routine activities [12] should be encouraged and reinforced. This would allow the protection of children that missed the routine age window and slow the build-up of susceptible children contributing to the risk of an epidemic in areas with circulating virus. While EPI programs should be flexible to ensure vaccination, efforts are needed to ensure an early first dose administration as a first priority.

In contexts where SIAs were implemented, coverage was low and far under the targeted 95%. Moreover, in many locations these activities were not implemented or often implemented late, without respecting the recommended interval between SIAs. For instance, in Katanga province, after the 2007 SIA few measles cases were reported. The next SIA was planned in 2010, but this was postponed [13] contributing to the causes of a large outbreak in 2010-2011 [14].

Often administrative coverage was higher than survey results. This was most significant for SIA, where all administrative estimates were higher than surveys results. Administrative estimates are often at national level and do not account for provincial or district differences. Population-based surveys can provide specific information on coverage and help target interventions. A model that considers both administrative and survey data has even been developed to characterize the performance of the activities leading to the estimated coverage and help to predict the effect of future vaccination activities [15]. This kind of exercise is very important to adequately assess the risk of outbreaks.

Despite several opportunities, a non-negligible proportion of children remained unvaccinated or had not received the recommended 2 doses. Conversely, for some children ORI provided the third or higher dose. Moreover, children not reached either by EPI program or by SIA were also less reached by ORI. These results highlight that children are not equally reached by vaccination activities and multiplying vaccine opportunities does not always imply that unvaccinated children are reached. Further work is needed to ensure that immunization activities reach unprotected children. And in case of limited resources, previous MCV vaccination activities and their coverage should be evaluated to better allocate resources and improve coverage.

These survey data are subject to limitations. First, surveys were conducted only in settings were measles control strategies were not efficient enough to avoid an outbreak, but where surveillance system detected an increase in measles cases. Vaccine coverage in these areas is likely to be different from places where no outbreak occurred or was detected during the last decade.

Second, although card confirmation is the preferred method for ascertaining coverage, this is not always possible and oral history is considered. For example, none of the children could show a vaccination card for SIA, and only an average of 16.9% of the children considered as vaccinated through EPI could show their vaccination card [range 1.8% - 36.2%]. Vaccination status regarding ORI was better documented as half of the children considered as vaccinated could show their vaccination card [range: 0.0% - 83.4%]. This proportion was generally high, except in Malawi and Maroua where quasi none of the vaccination status was confirmed by card. As a result, over-reporting or under-reporting of vaccination might have occurred, depending on the context and despite probing questions. However, previous studies in areas of high measles incidence have shown parental recall to be reliable [16].

Third, most of the surveys were conducted within a month after the end of ORIs. There is therefore low risk of recall bias for ORI coverage estimates, especially as there is less risk of card loss. Information on routine vaccination was collected for all age groups, i.e. for children up to 15 years of age. Recall bias, especially for older children for which vaccination in routine vaccination occurred long ago, might be expected. We therefore chose not to present EPI coverage for older age group but this likely resulted in an overestimation of the number of doses received in older age groups.

Finally, although standardized protocols and training of all surveyors was rigorously implemented across settings, there may be additional inaccuracies in the data related to individual interviewers and supervisors as well as the inaccuracies of population data used to select the samples.

It is also important to recognize that although population surveys are a rapid means to obtain a vaccine coverage estimate, without serological confirmation, estimates of coverage remain an inference.

## Conclusion

Population-based surveys are important to have reliable estimates of MCV coverage and can serve as a tool to assess different vaccine opportunities and to have a better understanding of coverage variations among age group and settings. Control pre-elimination targets were still not reached in the studied sites and might explain the occurrence of repeated measles outbreak in Sub-Saharan African countries. Furthermore, despite different vaccine opportunities, the number of unvaccinated or not fully vaccinated children was high in some settings. Strategies to better target hard-to-reach children are needed.

## Abbreviations

WHO: World Health Organization; MCV: Measles-containing vaccine; EPI: Expanded program on immunization; SIA: Supplementary immunization activities; ORI: Outbreak response immunization campaigns; MoH: Ministry of Health; MSF: Non-governmental organization Médecins sans Frontières; CAR: Central African Republic; DRC: Democratic Republic of Congo; CI: Confidence interval.

## Competing interests

The authors declare that they have no competing interests.

## Authors’ contributions

LG implemented surveys in DRC in 2011, conducted the second analysis of all datasets and drafted the manuscript; NC participated to the second analysis of all datasets and the interpretation of the results; AJG and EG helped to the data interpretation and to draft the manuscript; NH and FF helped to the conception of the surveys, the data collection and to draft the manuscript; AN helped in the data collection and data analysis in some of the surveys and to the data interpretation; KP conceived the surveys, participated in their design and coordination and helped to draft the manuscript; RFG conceived of the second analysis and helped to draft the manuscript. All authors read and approved the final manuscript.

## Pre-publication history

The pre-publication history for this paper can be accessed here:

http://www.biomedcentral.com/1471-2458/14/193/prepub

## Supplementary Material

Additional file 1: Table S1 Design, sample size and vaccination opportunities assessed in the population-based surveys, 2005-2011. Click here for file
