# Supplementary material for: Local discrepancies in measles vaccination opportunities: results of population-based surveys in Sub-Saharan Africa
Source: BMC Public Health. 2014 Feb 21;14:193. doi: 10.1186/1471-2458-14-193 (PMC3938072; doi:10.1186/1471-2458-14-193)
Supplement: Additional file 1: Table S1 — Design, sample size and vaccination opportunities assessed in the population-based surveys, 2005-2011. [file 1471-2458-14-193-S1.pdf]

Additional file: Design, sample size and vaccination opportunities assessed in the population-based surveys, 2005-2011.

Survey						Design			Sample size			Opportunities assessed			
**Country**	**Place**	**Context**	**Year**	**Target**	**Days after ORI**	**Sampling**	**Selection of children**	**Stratification**	**Hypothesis***	**Sampling plan**	**N**	**EPI**	**Last SIA**	**Previous ORI**	**MSF ORI**
Chad	N'Djaména	Urban	2005	6-59m	30 days	LQAS	spin the pen	-	-	25 lots of 65 children	1558	x			x
DRC	Matadi	Urban	2006	6m-15y	17 days	2-stage cluster	spin the pen	-	VC=65%; AP=2.5%	30 clusters of 24 households	2105	x			x
	Mbuji-Mayi	Urban	2006	6-59m	12 days	2-stage cluster	spin the pen	3 administrative levels	VC=65%; AP=3%	30 clusters of 24 households	3591	x	x		x
Cameroon	Maroua	Urban	2009	9m-15y	68 days	cluster	satellite-based	3 measles incidence levels	VC=65%/75%/85%; AP=7; deff=3	24/20/14 clusters of 22 children	2708	x		x	x
Chad	N'Djamena	Urban	2010	6m-15y	8 days	LQAS	GPS-based	-	-	28 lots of 65 children	1820	x		x	x
Malawi	Blantyre	Urban-Rural	2010	6m-15y	51 days	cluster	satellite-based	-	VC=80%; AP=5%; deff=4; 0.7 children per house	40 clusters of 35 households	2978	x	x	x	x
	Chiradzulu	Rural	2010	6m-15y	48 days		spin the pen	-			3106	x	x	x	x
	Thyolo	Rural	2010	6m-15y	32 days		spin the pen	-			3331	x	x	x	x
	Mangochi	Rural	2010	6m-15y	15 days		spin the pen	-			3402	x	x	x	x
	Lilongwe	Urban-Rural	2010	6m-15y	44 days		satellite-based	-			2517	x	x	x	x
	Mzimba	Urban-Rural	2010	6m-15y	62 days		spin the pen	-			3427	x	x	x	x
	Balaka and Machinga	Urban-Rural	2010	6m-15y	19 days		spin the pen	-			3232	x	x	x	x

DRC: Democratic Republic of Congo; CAR: Central African Republic

VC: vaccination coverage; AP: accepted precision; EPI: Expanded Program of Immunization; SIA: Supplementary immunization activity; ORI: Outbreak response immunization

*For all the studies α=0.05

Additional file (continuation): Design, sample size and vaccination opportunities assessed in the population-based surveys, 2005-2011.

Survey						Design			Sample size			Opportunities assessed			
**Country**	**Place**	**Context**	**Year**	**Target**	**Days after ORI**	**Sampling**	**Selection of children**	**Stratification**	**Hypothesis***	**Sampling plan**	**N**	**EPI**	**Last SIA**	**Previous ORI**	**MSF ORI**
DRC	Likasi city	Urban	2011	6m-15y	2 days	3-stage cluster	satellite-based	Health zone	Each strata: VC=90%; AP=5%	Each strata: 18 clusters of 16 compounds	3919	x	x		x
	Lubumbashi	Urban	2011	6m-15y	11 days	3-stage cluster	satellite-based	Measles incidence Administrative	In each pair of strata: VC=90%; AP=5%	In each pair of strata: 24 clusters of 19 compounds	6172	x	x		x
	Kapolowe	Rural	2011	6m-15y	1 day	3-stage cluster	spin the pen / satellite	-	VC = 80%; AP=5%; 2 eligible in each compound	30 clusters of 10 compounds	968	x	x		x
	Kambove	Rural	2011	6m-15y	4 days	3-stage cluster	spin the pen	-	VC = 80%; AP= 5%; 2 eligible in each compound	30 clusters of 10 compounds	1128	x	x		x
	Kasenga	Rural	2011	6m-15y	95 days	3-stage cluster	spin the pen	Low/High administrative VC	VC=45%/70%; AP=5%; 2 eligible in each compound	31/26 clusters of 13 compounds	2009	x	x		x
	Malemba-Nkulu	Rural	2011	6m-15y	38 days	3-stage cluster	spin the pen	Low/High administrative VC	VC=50/90%; AP=8%/5%	Each strata: 30 clusters of 10 compounds	1240	x	x		x
	Kipushi	Rural	2011	6m-15y	117 days	3-stage cluster	spin the pen	-	VC=81%; AP=5%; 2 eligible in each compound	30 clusters of 13 compounds	1432	x	x		x
Burundi	Kirundo	Rural	2011	6m-15y	24 days	3-stage cluster	random	-	VC=90%; AP=5%	30 clusters of 12 compounds	901	x			x
Chad	Moïssala 1	Rural	2011	9m-5y	48 days	3-stage cluster	spin the pen	-	VC=75%; AP=6%; deff=3	35 clusters of 23 compounds	1502	x		x	
	Moïssala 2	Rural	2011	9m-5y	48 to 61 days	3-stage cluster	random	-	VC=60%; AP=6%; deff=2	35 clusters of 16 compounds	1273	x			x
CAR	Kabo	Urban	2011	6m-15y	> 31 days	2-stage cluster	spin the pen	2 administrative levels	VC=80%; AP=6%; deff=3	30 clusters of 20 compounds	480	x		x	x
	Batangafo	Urban-Rural	2011	6m-15y	38 days	3-stage cluster	spin the pen	-	VC=80%; AP=6%; deff=4	30 clusters of 30 compounds	2797	x		x	x

DRC: Democratic Republic of Congo; CAR: Central African Republic.

VC: vaccination coverage; AP: accepted precision; EPI: Expanded Program of Immunization; SIA: Supplementary immunization activity; ORI: Outbreak response immunization

*For all the studies α=0.05
